# Supplementary material for: The Prevalence of Immediate Hypersensitivity Reactions to the BNT162b2 mRNA Vaccine against SARS-CoV-2: Data from the Vaccination Campaign in a Large Academic Hospital
Source: Vaccines (Basel). 2023 Apr 27;11(5):903. doi: 10.3390/vaccines11050903 (PMC10223765; doi:10.3390/vaccines11050903)
Supplement: Supplementary file 1 [file vaccines-11-00903-s001.zip › vaccines-2346102-supplementary.pdf]

Supplementary data. Questionnaire.

- Name
- Surname
- Sex (Female/Male)
- Age
- Weight
- Height
- In which facility do you work?
- Do you suffer for any allergic disease? (Yes/No)
- COVID-19 vaccine administration dose (first or second dose)
- Date of first dose of COVID-19 vaccine
- Date of second dose of COVID-19 vaccine
- Did you have a suspected adverse reaction to COVID-19 vaccine? (Yes/No)
- Did the first reaction occur on the day of vaccination? (Yes/No)
- Onset date of first symptoms
- Date symptoms ended (if finished)
- Have you been to the emergency room because of this reaction? (Yes/No)
- Which of these symptoms have you had? Please, indicate the duration of each symptom:
1 day, 2-3 days, 4-7 days, 7-14 days, 14-21 days.
o Local reactions at the site of injections)
o Fatigue
o Headache
o Muscle pain
o Joint pain
o Fever (TC <38°)
o Fever (TC >=38°)
o Swelling and soreness of axillary lymph nodes (on the vaccination side)
o Chills
o Skin rash (localized)
o Diffuse rash
o Anxiety/panic attack
o Feeling of fainting
o Loss of consciousness
o Sudden abdominal pain
o Insomnia
o Diarrhea
o Nausea
o Vomiting
o Swelling of the face
o Swelling of the tongue or the throat
o Transient facial paralysis
o Hypotension (sudden drop in blood pressure)
o Sweating
o Tachycardia (fast heart rate)
o Oppression or chest pain
o Shortness of breath
o Other
- If you checked "Other" specify,
- If you have had a fever report the maximum body temperature recorded
- To date the reaction is:

o fully resolved,
o resolved but with some aftereffects,
o is getting better,
o has remained unchanged/worsened,
o I don’t know

- Have you had other vaccinations in the 4 weeks prior to receiving the COVID-19 vaccine dose? If yes, which vaccination?
- Have you ever had a reaction after receiving a vaccine? If yes, which ones?
- Have you had COVID-19? (YES/NO)
- Have you had any symptoms? (YES/NO)
- Did you have to take medication to relieve the symptoms? (YES/NO)
- Do you have any other observations you wish to report?
